# Prevalence and Associated Factors of Intestinal Parasitic Infections among Food Handlers in Mettu Town, Southwest Ethiopia

**DOI:** 10.1155/2021/6669734

**Published:** 2021-02-18

**Authors:** Solomon Yeshanew, Melaku Tadege, Abdulhakim Abamecha

**Affiliations:** ^1^Department of Biology, Debre Markos University, P.O. Box 269, Debre Markos, Ethiopia; ^2^Department of Statistics, Bahir Dar University, Bahir Dar, P.O. Box 79, Amhara, Ethiopia; ^3^Department of Statistics, Injibara University, P.O. Box 40, Amhara, Ethiopia; ^4^Department of Biomedical Sciences, Mettu University, P.O. Box 318, Mettu, Ethiopia; ^5^Department of Medical Laboratory Sciences, Jimma University, P.O. Box 378, Jimma, Ethiopia

## Abstract

Food handlers are important sources of intestinal parasitic infection to the public and mass catering service areas. Several reports worldwide particularly in developing countries showed a high prevalence of intestinal parasitic infections among these groups of individuals. In an attempt to determine the prevalence of intestinal parasites, a cross-sectional study was carried out among food handlers in food establishment areas of Mettu town, Southwest Ethiopia. To collect sociodemographic characteristics of the study participants, a structured questionnaire and physical observation were employed. Stool specimens collected from each food handlers participated in the study were then examined using light microscopy of the wet mount followed by formol-ether concentration methods to see infection status. A total of 139 food handlers were enrolled in the study. Majority of them were females 89 (64%), and 124 (89.2%) of them had not taken any training related to food handling and preparation. Sixty-two (44.6%) of the study participants were diagnosed with one or more intestinal parasites. The chi-square test showed that intestinal parasite infection was found associated with personal hygiene (*p* ≤ 0.0001), nail trimming status (*p* ≤ 0.0001), and hair cover status (*p* ≤ 0.040). The multivariable logistic regression indicated that the risk to be infected with intestinal parasites were related with older age (*p* ≤ 0.032), food handlers who had no hand wash practices (*p* ≤ 0.033), who had no food handling and preparation training (*p* ≤ 0.005), poor personal hygiene (*p* ≤ 0.0001), who had not taken regular medical checkup (*p* ≤ 0.008), and whose nail was not trimmed (*p* ≤ 0.0001). The most abundant intestinal parasite identified was *G. lamblia* (24, 26.7%) followed by *E. histolytica/dispar* (22, 24.4%), and *A. lumbricoides* (15, 16.7%). Twenty-eight (45.2%) study individuals were also found with mixed infections. The study thus revealed a high prevalence of intestinal parasitic infection among apparently healthy food handlers in food establishments of the study area. Mass drug administration for all food handlers working in food establishment areas of the town coupled with health education and training should be initiated urgently.

## 1. Introduction

Foodborne diseases continue to be a major global health problem and are the leading causes of morbidity and mortality in developing countries [1]. The World Health Organization (WHO) estimated 30% of the population in developed countries, suffers from foodborne diseases each year, whereas in developing countries, up to two million deaths are estimated per year, and more than 70% cases of diarrheal diseases are associated with the consumption of contaminated food [2].

Food handlers are an important source of contamination either as carriers of pathogens or through poor hygienic practices [3]. Subsequently, several reports showed a high prevalence of intestinal parasitic infections among food handlers in different parts of the world [4–7]. CDC also reported that approximately 20% of food-related infection is due to food handlers. A similar study in the USA also suggested that improper food handler practices contributed to approximately 97% of foodborne illnesses in food service establishment areas and homes [8].

In Ethiopia, the rate of intestinal parasitic infection among food handlers were conducted and ranged from 29 to 72% with different reports on the predominant species of parasites and hygiene practices [9–13]. The importance of food handlers as threats in the transmissions of intestinal parasitic infections has been due attention by several authors [5, 6, 10, 11]. However, there is a scarcity of reported data on food handlers being identified as potential sources of intestinal parasitic infection from several parts of the country. According to the clinical data generated from Mettu Karl Hospital and a report from the Health Office of Mettu town, Southwestern Ethiopia, more than 50% of the population was estimated to be positive for intestinal parasites. Hence, an attempt is made in the present study to determine the prevalence of intestinal parasites and associated predisposing factors among food handlers serving in food establishment areas of the town.

## 2. Methods

### 2.1. Study Design and Area

A cross-sectional study was carried out among food handlers working in food establishment areas of Mettu town between March and June 2016. Mettu is a capital town of Mettu district and Ilu Aba Bora zone. The town is located approximately 600 km southwest of the capital Addis Ababa, Ethiopia. The district covers an area of 1452 km^2^ which lies between 8°6′ and 8°31′N latitude and 35°10′ and 35°50′E longitude plus an altitude range from 1000 to 2027 m above sea level. According to the 2013 national census, the number of inhabitants was estimated to be 54,792 (22,857 male and 31,935 female).

### 2.2. Study Population and Sample Size

Food handlers working at different kitchens of food service establishments and who were directly involved with food preparation and serving food to customers in the town were enrolled in the study. A total of 139 study subjects were selected randomly from 37 different cafeterias, hotels, butcher shops, restaurants, and mass catering serving establishments of the town. The sample size was calculated using single population proportion formula as described by Daniel (1999) [14] considering 95% confidence of interval, 5% margin of error, and prevalence rate of 44.1% of the previous studies [9].

### 2.3. Specimen Collection and Transport

Stool samples were collected from each food handler using clean stool cups by professional and trained medical laboratory technicians and transported into the Medical Laboratory Section of Mettu Karl hospital within half an hour.

### 2.4. Microscopic Examination of Stool

Stool specimen from all study participants were investigated using light microscopy of the direct wet mount followed by formol-ether concentration sedimentation methods according to the WHO guideline [15] in the hospital's parasitology research laboratory room.

### 2.5. Statistical Analysis

Analysis of the data was undertaken using SPSS version 20 software. Percentage and frequency distribution were used to determine the prevalence of intestinal parasitic infection. To see the association between the independent variables with parasitic infections status, the chi-square test was applied. Similarly, the effect of each exposure variables on the outcome variable was assessed using binary logistic regression analysis and *p* value <0.05 was considered as a statistically significant level of intestinal parasitic infection.

## 3. Results

A total of 139 food handlers were selected randomly, and all of them agreed to participate in the study with a response rate of 100%. Sixty-two (44.6%) of the food handlers participated in the study were found to be positive for various types and species of intestinal parasites. Eighty-nine (64%) of the participants were females, and 50 (36%) were males. Majority of the study participants had less than a year of experience in food handling and preparation. Only 15 (10.8%) of the food handlers enrolled in the study were certified in food preparation and handling training. Twenty-five (18.0%) had medical checkup practice, and 79 (56.8%) did not trim their fingernail. Among the 79 (56.8%) fingernail nontrimmed study participants, 55 (88.7%) were found positive with intestinal parasites. As such, the chi-square test approved that there was a statistically significant association between fingernail status and prevalence of intestinal parasitic infection (*X*^2^ = 46.352, *p* ≤ 0.0001) at 5% significant levels. The statistics of the number of participants regard to have proper hand wash practice, and medical checkup were far from adequate. Concerning personal hygiene of the study participants, 27 (19.4%) , 61 (43.9%), and 51 (36.7%) were good, moderate, and poor status respectively. Around two-thirds (66.1%) of intestinal parasitic infections were found among study subjects categorized under poor status. Regard to hair cover, 100 (71.9%) of them had not used. Therefore the study deduced that intestinal parasite infections were highly associated with personal hygiene (*p* ≤ 0.0001), nail trimming status (*p* ≤ 0.0001), and hair cover status (*p* ≤ 0.040) (Table 1).

Binary logistic regression analysis was conducted to determine major responsible factors for a high prevalence of intestinal parasitic infections in food handlers enrolled in the study. From univariate analysis, food handlers who had no hand wash practice, poor personal hygiene, and those who did not trim their nails were a predictor of intestinal parasitic infection. Since univariate analysis ignores the relationship between independent variables, we used multivariable analysis procedure for producing a valid conclusion about the population parameter. Therefore, multivariable logistic regression output stated that when the age of the food handlers increased by a year, the odds ratio of having intestinal parasitic infection were 1.176, meaning the chance of infection with intestinal parasites were increased by 17.6% (AOR = 1.1760 (1.0140, 1.3640), *p* ≤ 0.0320). Food handlers who had a regular hand wash practice decreased the possibility to be infected by 0.778% (AOR = 0.2220 (0.0560, 0.8830), *p* ≤ 0.0330). Likewise, study participants who acquired food handling and preparation training have decreased the chance of getting intestinal parasitic infections by 97.9% (AOR = 0.0210 (0.0010, 0.3080), *p* ≤ 0.0050). Similarly, participants of the study who had medical checkup decreased the probability of infection by 95.9% (AOR ≤ 0.0410 (0.0040, 0.4340), *p* ≤ 0.0080) (Table 2).

Sixty-two intestinal parasitic cases were diagnosed from study participants with the most frequently identified parasite *G. lamblia* which was found in 24 (26.7%) food handlers followed by *E. histolytica/dispar* (22, 24.4%) and *A. lumbricoides* (15, 16.7%). From the sixty-two positive food handlers for intestinal parasites, 46 (74.2%) had protozoan and 44 (71.0%) diagnosed with helminths. However, 28 (45.2%) of them were found with mixed infections. Nine (14.5%) were positive for *E. histolytica/dispar* and hookworm, and 6 (9.7%) were by *E. histolytica/dispar* and *A. lumbricoides*. Similarly, *G. lamblia* with *T. trichiura* and *G. lamblia* with *A. lumbricoides* were also found concurrently with the prevalence of 8.2% in each case (Table 3**)**.

## 4. Discussion

Among food handlers working in food service establishment areas of Mettu town enrolled in the present study, nearly half (44.6%) of them had been developing an intestinal parasitic infection. The finding was higher as compared with previous reports from southern Ethiopia (36%) [16], Ghana (21%) [6], Saudi Arabia (31.9%) [17], and Kenya (24%) [18]. However, it was in line with the report from Brazil (47.1%) [19], Venezuela (48.7%) [20], and Jordan (48.0%) [21]. Similarly it was consistent with other investigations from elsewhere in Ethiopia such as Mekele (49.4%) [10], Bahir Dar (41.1%) [12], Yebu (44.1%) [9], and Addis Ababa (45.3%) [11]. In contrast to the present findings, reports from Jos, Nigeria (55.9%) [22], from Hawassa, Ethiopia (63%) [23], and Abeokuta, Nigeria (97%) [24], were greater. This inconsistency might arise due to sample size and environmental variation, various habits of personal hygiene, and different settings of sociodemographic characteristics of the target population.

The study showed that intestinal parasitic infection was associated with nontrimmed fingernail status output, but other sociodemographic characteristics such as gender were not a predictor for infection. A similar study from Addis Ababa [11] was found in agreement with the present study. The study stated that hand wash habit, medical checking up status, and training were the most responsible factors of intestinal parasitic infection which was in line with reports from Yebu [9], Mekele [10], Nigeria [22], and southern Ethiopia [16]. The dominant parasite identified in the present study was *G. lamblia* 24 (26.7%) followed by *E. histolytica/dispar* 22 (24.4%). Fifteen (16.7%) and 12 (13.3%) of the study participants were found positive for *A. lumbricoides* and Hookworm infection, respectively. The report from Addis Ababa [11], southern Ethiopia [16], Bahirdar [12], and Mekele [10] showed that *E. histolytica/dispar* and *G. lamblia* were the most abundant intestinal parasites diagnosed from study food handlers. However, a contrast finding from Yebu [9] and Gonder [25] reported *A. lumbricoides* and *T. trichiura* were detected at a high rate. This discrepancy could be due to the variation in the ecology and climatic differences of the study areas and the town level variations. Twenty-eight (20.1%) food handlers enrolled in the study were diagnosed with mixed infection. Studies from Addis Ababa [11], Southern Ethiopia [16], Mekele [10], and Bahir Dar [12] similarly reported two or more intestinal parasites found concurrently on an individual.

## 5. Conclusions

Prevalence of intestinal parasites among food handlers in food service areas of Mettu town was 44.6%. Poor handwashing habit, older age, nontrimmed fingernail, lack of proper training, and absence of regular medical checkup were the major predisposing factors of harbouring intestinal parasites among the study participants. We recommend an urgent need for mass drug administration and food preparation and handling training to all food handlers and managers of food service areas in the study locality.

## Figures and Tables

**Table 1:** Sociodemographic characteristics with respect to prevalence of intestinal parasites among food handlers in food establishment areas of Mettu town.

Variables		Total examined *n* (%)	Infection status *n* (%)		Chi-square (*X*^2^)	*p* value
			Yes	No		
Gender	Male	50 (36)	19 (30.6)	31 (40.3)	1.378	0.240
	Female	89 (64)	43 (69.4)	46 (59.7)		
Personal hygiene	Good	27 (19.4)	3 (4.8)	24 (31.2)	44.320	0.0001
	Moderate	61 (43.9)	18 (29.0)	43 (55.8)		
	Poor	51 (36.7)	41 (66.1)	10 (13.0)		
Hand wash habit	Yes	57 (41)	20 (32.3)	37 (48.1)	3.541	0.060
	No	82 (59)	42 (67.7)	40 (51.9)		
Training certified	Yes	15 (10.8)	6 (9.7)	9 (11.7)	0.144	0.704
	No	124 (89.2)	56 (90.3)	68 (88.3)		
Nail status	Trimmed	60 (43.2)	7 (11.3)	53 (68.8)	46.352	0.0001
	Not trimmed	79 (56.8)	55 (88.7)	24 (31.2)		
Medical checkup	No	114 (82)	55 (88.7)	59 (76.6)	3.401	0.065
	Yes	25 (18)	7 (11.3)	18 (23.4)		
Hair cover	No	100 (71.9)	50 (80.6)	50 (64.9)	4.199	0.040
	Yes	39 (28.1)	12 (19.4)	27 (35.1)		
Gowan cover	No	42 (30.2)	18 (29.0)	24 (31.2)	0.074	0.785
	Yes	97 (69.8)	44 (71.0)	53 (68.8)		
Work experience	<2 years	76 (54.7)	32 (42.1)	44 (57.9)	3.498	0.329
	2–5 years	42 (30.2)	23 (54.8)	19 (45.2)		
	5–10 years	16 (11.5)	6 (37.5)	10 (62.5)		
	>10 years	5 (3.6)	1 (20)	4 (80)		

**Table 2:** Binary logistic regression analysis of intestinal parasitic infections and corresponding factors among food handlers at Mettu town food establishment areas.

Variable	Category	Univariate analysis		Multivariable analysis	
		COR (95% CI)	*p* value	AOR (95% CI)	*p* value
Age		1.0110 (0.9980, 1.0240)	0.1010	1.1760 (1.0140, 1.3640)	0.0320
Hand wash habit	Yes	1.8500 (1.0740, 3.1870)	0.0270	0.2220 (0.0560, 0.8830)	0.0330
	No (ref)				
Training certified	Yes	1.5000 (0.5340, 4.2140)	0.4420	0.0210 (0.0010, 0.3080)	0.0050
	No (ref)				
Nail status	Not trimmed	7.5710 (3.4420, 16.6530)	0.0001	10.4150 (2.9380, 18.9240)	0.0001
	Trimmed (ref)				
Medical checkup	Yes	1.0730 (0.7430, 1.5490)	0.7080	0.0410 (0.0040, 0.4340)	0.0080
	No (ref)				
Gowan cover	No	1.3330 (0.7240, 2.4570)	0.3560	2.9160 (0.6740, 12.6210)	0.1520
	Yes (ref)				
Gender	Male	1.6320 (0.9220, 2.8880)	0.0930	0.8790 (0.2330, 3.3160)	0.8480
	Female (ref)				

**Table 3:** Type and prevalence of intestinal parasites isolated from stool specimens of food handlers from food establishment areas of Mettu town.

Intestinal parasite	Frequency	Percentage
Protozoan		
*E. histolytica/dispar*	4	6.4
*G. lamblia*	14	22.6
Helminths		
*A. lumbricoides*	4	6.4
*T. trichiura*	4	6.4
*H. nana*	2	3.2
*Hookworm*	3	4.8
*S. mansoni*	1	1.6
*Taenia* spp.	2	3.2
Mixed infection		
*E. histolytica/dispar* and *A. lumbricoides*	6	9.7
*E. histolytica/dispar* and *Hookworm*	9	14.5
*E. histolytica/dispar* and *S. mansoni*	1	1.6
*E. histolytica/dispar* and *T. trichiura*	2	3.2
*G. lamblia* and *T. trichiura*	5	8.2
*G. lamblia* and *A. lumbricoides*	5	8.2
Total	**62**	**100**

## Data Availability

The raw data used to support the findings of this study are available from the corresponding author upon request.

## Abbreviations

AOR: Adjusted odd ratio
CDC: Center for disease control
CI: Confident interval
COR: Crude odd ratio
SPSS: Statistical package for social sciences
USA: United States of America
WHO: World Health Organization.
